# Health literacy strengths and limitations among rural fishing communities in Egypt using the Health Literacy Questionnaire (HLQ)

**DOI:** 10.1371/journal.pone.0235550

**Published:** 2020-07-16

**Authors:** Wagida A. Anwar, Nayera S. Mostafa, Sally Adel Hakim, Dalia G. Sos, Dena A. Abozaid, Richard H. Osborne

**Affiliations:** 1 Department of Community, Environmental and Occupational Medicine, Faculty of Medicine, Ain Shams University, Cairo, Egypt; 2 Centre for Global Health and Equity, Faculty of Health, Design and Arts, Swinburne University of Technology, Melbourne, Australia; Jawaharlal Nehru University, INDIA

## Abstract

**Introduction:**

Health literacy is an important determinant of health. The aim of this study was to use a multi-dimensional measurement tool to describe the health literacy of people living in a fishing community in northern Egypt.

**Methods and analysis:**

Data were collected from 436 people (fisherman and their families), using the Health Literacy Questionnaire (HLQ), which includes 9 scales. Effect sizes (ES) for standardized mean differences estimated the magnitude of difference between demographic groups.

**Results:**

The mean age of participants was 42 years, 50% were male, 42% were working in the fishing sector, 17.9% had access to the Internet and 36.8% were illiterate. Male participants showed higher capabilities in scales *3*. *Actively managing my health* and *4*. *Social support for health* (ES = 0.21 and 0.27, respectively). In comparison to other occupations, fishing occupation had a negative impact on scale *7*. *Navigating the healthcare system* (ES -0.23). Also, higher educational level was associated with higher HLQ indicators. Across all scales, scale *2*. *Having sufficient information to manage my health* showed the lowest mean (SD) score; 2.23 (0.76) indicating that most people reported they didn’t have enough information.

**Conclusions:**

This study has revealed that fishermen and their families have a wide range of health literacy difficulties which are likely to have profound negative effects on health behavior and health outcomes.

## Introduction

Health literacy is defined as a person’s ability to access, understand, appraise, remember/retrieve, and use information about health and health services [1]. It is a concept that is broader than health education because health literacy addresses environmental, political and social factors that influence an individual’s ability to engage with health information and health services. Health literacy is influenced by the strengths and limitations of individuals and of communities. Some of them are related to the level of education of the population and standard of healthcare services [1]. In order to improve health inequities in a community, the diversity of health literacy strengths and limitations of individuals must be assessed, and interventions designed and delivered to address this diversity [2]. Furthermore, for an organization or health service to be health literacy responsive, it needs to understand the health literacy strengths and limitations of the people in that community, and to facilitate access to health information and services to all people, regardless of their health literacy limitations [2,3].

There is limited research on health literacy in Arab countries. A study conducted in Saudi Arabia using a word recognition test found that 57.4% of Saudi Arabians had difficulty recognizing health-related words [4]. In Egypt, a study explored health-related reading and numeracy (i.e., functional health literacy) among elderly caregivers in the Geriatrics Medicine Department of Ain Shams University (ASU) Hospitals. Findings indicated about 75% of the participants were classified as having limited functional health literacy. Another study showed that lower functional health literacy was associated with higher frequency of hospitalization, longer hospital stays, and lower health-related quality of life [5]. A study among people attending outpatient clinics at Ain Shams University Hospitals found that half of the participants did not have enough reading, writing and information processing abilities to effectively participate in their own care [6].

While HL is a challenge for people attending healthcare services in cities (like capitals and developed areas), people working in hazardous labor industries in rural regions potentially have greater personal health challenges, particularly given limited access to high quality healthcare services. One of the oldest rural occupations is fishing [7]. Fishermen are exposed to cold, wind, rough seas, hard physical labor, and they frequently sustain injuries during their work [8]. The International Labour Organization stated that in developing countries, many communities, such as fishing communities, are in remote areas with poor living conditions. Generally, people working in small-scale fishing enterprises are exposed to risks such as bad weather, onboard fire hazards, inadequate boat construction standards, loss of power, lack of accessible shelters, and isolation through inadequate radio communication facilities [9].

Alongside the hazards of the fishing occupation, fishermen are at high risk for chronic diseases due to an unhealthy lifestyle. A Danish study indicated that fishermen were at increased risk of hospitalization for lifestyle-related diseases such as diabetes, heart diseases, bronchitis, emphysema, cancer of the lung, alcohol-related liver diseases, and Raynaud’s syndrome [10]. Other studies showed that fishing is an occupation with high incidence of musculoskeletal disorders and injuries, and even increased risk of fatalities [11–13].

Globally, fishing communities are highly disadvantaged because they tend to be denied a range of healthcare services [14]. In Egypt, a study found that fishermen in Alexandria were at excessively high risk of musculoskeletal disorders, auditory complaints, sunburn and injuries. It was found that fishermen tended to spend long hours working at sea and were suffering from psychological stress, job instability, and were infrequent users of personal protection equipment [15]. A study in Bangladesh reported that the fishermen were a disadvantaged and neglected group [16]. Disadvantaged populations are defined as *“those persons who*, *for a number of reasons*, *see their capacity to take advantage of opportunities diminished in comparison with other groups*.*”* [17]. These populations are characterized by their inability to participate fully in social and economic activities, as well as their low decision-making ability, high social exclusion, and reduced access to essential goods and services such as health care [18].

There is a need for health promotion and education initiatives to raise awareness in fishing communities about health promotion and the management of chronic diseases [19]. This study could play a role in the required health promotion, as it aimed to describe the health literacy of a community of fishermen and their families living around Borollos Lake in Egypt. Borollos Lake is located in Kafr El Sheikh, one of Delta region's governorates, has a large group of inhabitants working as fishermen. Multi-dimensional Health Literacy Questionnaire (HLQ) [20] was used to gain an in-depth understanding of the health literacy strengths and limitations of community members to guide potential development of local and regional interventions.

## Methods

### Study design

The study is part of a larger project taking place in the rural Borollos Lake region that aims to develop a comprehensive and holistic description of the fishing community, their health problems, and the current health and environmental risk factors. This health literacy study uses the OPtimising HEalth LIteracy and Access (Ophelia) process to intervention co-design and implementation [21]. This paper documents the quantitative data collection for the first of the three phases of the Ophelia process. The first phase involves a cross-sectional survey to describe the health literacy profile of the Borollos Lake fishing community, which is a fundamental needs assessment activity.

### Setting and participants

Borollos Lake is surrounded by 5 sub-regions. The majority of their inhabitants work in fishing. The sample of the study comprised fishermen and their families, who were recruited from five villages representing the five sub-regions around Borollos Lake. One village from each sub-region was selected based on the perceptions of community leaders regarding their need for community health promotion interventions. Participant selection criteria were deliberately unrestrictive but required that participants be over 18 years of age and be able to provide written informed consent following the provision of an explanation of the project’s rationale and procedures. With the aid of the non-governmental organizations and fishermen syndicates, announcements were done before the research team arrived to the village to arrange for the meetings with the fishermen and their families at one of the non-governmental organizations settings. Although the study procedure used convenience sampling, all fishermen and their families that were were invited to participate in the study did participate. Recruitment of participants took place in the period from January–May 2018.

### Data collection

Participants were interviewed by a health worker or research assistant. The HLQ was administered verbally. Socio-demographic data collected included age, sex, living alone or with others, Internet usage, family income, occupation (fisherman or other), educational attainment (illiterate, primary level or above primary level).

The HLQ consists of nine scales each with between 4 and 6 items. The nine scales are mentioned in Table 1.

**Table 1:** HLQ scales.

1.	Feeling understood and supported by healthcare providers
2.	Having sufficient information to manage my health
3.	Actively managing my health
4.	Social support for health
5.	Appraisal of health information
6.	Ability to actively engage with healthcare providers
7.	Navigating the healthcare system
8.	Ability to find good health information
9.	Understand health information well enough to know what to do

The HLQ has been widely used, and validity testing of the English HLQ has been undertaken in several communities [22,23] and translated into more than 30 languages. The HLQ was specifically developed and for surveys and intervention evaluation as well as for exploring the health literacy needs of individuals and communities [20,24]. Validity testing of translated versions has been undertaken in several countries and these studies have contributed to the network of evidence for valid decisions using HLQ data in these cultural contexts [25–29]. A license to use the previously-developed Arabic version of the HLQ was obtained. As formerly mentioned, the HLQ was validated and translated to many languages including Arabic. The Egyptian team undertook qualitative work in the form of local Egyptian Arabic linguistic and cultural adaptation to ensure all items were culturally appropriate and matched the original English HLQ item intent descriptions in the Translation Integrity Protocol [30,31]. The adaptation process included consensus meetings to ensure concepts and language elements were appropriate for the population and the item meaning, as closely as was possible, matched the original items. We found that all of the HLQ scales were relevant and easily understood in our context. A pre-study test was conducted with 12 participants from the Borollos Lake region. To improve readability only five changes were made to the questionnaire. Two specific Egyptian Arabic words were substituted for Arabic words that were less familiar to this population, whereas the other changes related to choice of a less complex word, replacement of an idometic expression with a literal expression and one word that was a better respresentation of the strength of an English expression [31]. For example, “healthcare provider” was changed to a rendition of “any professional who help take care of your health (for example doctor, nurse,.)” to maximise understanding. Also, “choose the healthcare provider you should see” was changed to “decide the specialty that suits your complaint” because the literation more closely matched the English intent.

The items in the first five HLQ scales have four response options scored 1 to 4: strongly disagree (1), disagree (2), agree (3) and strongly agree (4). Scales 6–9 have five response options (scored 1–5) Cannot do or usually difficult (1), very difficult (2), quite difficult (3), easy (4), and very easy (5). A scale score is devised by summing the item scores within each scale and dividing by the number of items in the scale, hence a scale score of 3 on the difficulty scale indicates and individual rated most items as quite difficulty. All scales have composite reliability from 0.8 to 0.9 [13] and the 8-scale factor structure and other strong psychometric properies have been confirmed in several languages [31].

### Statistical analysis

HLQ scales and socio-demographic data were analyzed using SPSS Version 21. Descriptive statistics described socio-demographic data. For all HLQ scales, responses covered the full range of the scales with modest or no floor effects but assumptions of normal distribution were not met. Scales 1 and 6–9 also violated homogeneity of variances. Hence robust analysis of variance (ANOVA) using the Welch method was used. Where required, post hoc testing was undertaken using the Games-Howell method of multiple mean comparisons. Effect sizes (ES) for standardized differences in means between demographic groups were calculated using Cohen’s d (calculated as the difference between the two means, divided by the pooled standard deviation (SD) of both means). The ES is interpreted as follows: ‘small’ ES >0.20–0.50, ‘medium’ ES 0.50–0.80, and ‘large’ ES >0.80 [32]. As was an exporatory, the first in Egypt with the HLQ and non-population-based study, no *a priori* sample size calculation was undertaken. Whenever suitable, 95% confidence intervals were calculated. A p-value of <0.05 was assumed for statistical significance.

## Results

A total of 436 participants were recruited from 5 villages (61 to 103 per village). The mean (SD) age of participants was 42 (15) years, range 18 to 89, with 65.2% of participants aged under 50 years (44% were in the middle-aged group (20–40 years)). Males comprised 50% of the sample, 37.1% were illiterate, and 42.4% were working actively as fishermen (see Table 2).

**Table 2:** Socio-demographic characteristics for overall sample (fisherman and their families).

	n	%
Age*		
< 50 yrs	285	(65.3)
≥ 50 yrs	151	(34.7)
***Sex***		
Male	216	(49.5)
Female	220	(50.5)
***Education***		
Illiterate	162	(37.1)
Primary level only	151	(34.6)
Higher than primary (N.B: only 16 of them finished university degree)	123	(28.3)
***Occupation***		
Fishermen	185	(42.4)
Housewives	184	(42.2)
Other (employees, skilled workers and students)	67	(15.3)
Income**		
≤ 2000L.E/month	385	(88.3)
>2000L.E/month	51	(11.7)
***Internet usage***		
Use Internet(at least once daily, weekly, monthly)	78	(17.9)
Never/rarely	358	(82.1)

*50 years is the median age of the study participants.

**2000 L.E. is equivalent to USD110 in 10 January 2019. It is the median of the income of the study participants.

As shown in Table 3, 78% of the fishermen worked on fishing boats far off shore. Exposure to extreme temperature as well as to noise and humidity were common occupational exposures.

**Table 3:** Occupational characteristics of fishermen included in the study (n = 185).

	n	%
***Exposure to occupational hazards***		
Noise	103	(55.7)
Extreme temperature	146	(78.9)
Awkward body postures	82	(44.3)
Accidents and injuries	51	(27.5)
Shift work (day / night)	99	(53.5)
***Duration of fishing career (years)***		
Median (IQR*) = 30 (25)		
***Work location*:**		
Far off shore	145	(78.3)
Near lake shores	40	(21.7)

*IQR: Interquartile Range

For HLQ scales 1–5 (see Table 1) (range 1 to 4), the highest mean (SD) score was seen for *4*. *Social support for health* (mean 2.9 (0.69)). Given how the items/scales are rated (1. strongly disagree to 4. strongly agree), this score indicated that most people agreed that they had social support. The lowest score was for *2*. *Having sufficient information to manage my health* (mean 2.2 (0.76)), followed by scales 3. and 5. that covered managing health and critical appraisal (2.37 for both) indicating that most people disagreed that they had sufficient information, could manage or appraise health information. For the last 4 scales (1. cannot do to 5. very easy), the highest score was for *6*. *Ability to actively engage with healthcare providers* (mean 3.5 (0.96)), indicating that most people scored between 2 and 3. The lowest score was for *8*. *Ability to find good health information* (mean 2.7 (1.1)) where most people scored from difficult to very difficult (see Table 4).

**Table 4:** Health Literacy Questionnaire (HLQ) scores for overall sample (fisherman and their families).

HLQ Scale	Mean (SD) [95% CI]
	Range 1 (lowest) 4 (highest)*
1. Feeling understood and supported by healthcare providers	2.51 (0.83) [2.43–2.58]
2. Having sufficient information to manage my health	2.23 (0.76) [2.16–2.30]
3. Actively managing my health	2.37 (0.75) [2.30–2,44]
4. Social support for health	2.95 (0.69) [2.89–3.03]
5. Appraisal of health information	2.37 (0.79) [2.29–2.44]
	Range 1 (lowest) 5 (highest)**
6. Ability to actively engage with healthcare professionals	3.50 (0.96) [3.41–3.59]
7. Navigating the healthcare system	3.11 (1.03) [3.01–3.21]
8. Ability to find good health information	2.78 (1.10) [2.67–2.88]
9. Understand health information well enough to know what to do	3.26 (0.88) [3.18–3.35]

Abbreviations = SD Standard Deviation, CI Confidence Interval

* 1 = strongly disagree, 2 = disagree, 3 = agree, 4 = strongly agree

** 1 = Cannot do or usually difficult, 2 = very difficult, 3 = quite difficult, 4 = easy, and 5 = very easy

Table 5 shows patterns of HLQ scores according to socio-demographic status. Differences (expressed as ES) provide an indication of the magnitude of differences. Differences were observed between age groups where those younger than 50 years reported slightly higher scores than those older than 50 years for *2*. *Have sufficient information to manage my health (ES -0*.*19)*, *5*. *Appraisal of health information (ES-0*.*22)*, *8*. *Ability to find good health Information(ES-0*.*31) and 9*. *Understand health information well enough to know what to do* (ES -0.27).

**Table 5:** Association between HLQ scores and socio-demographic characteristics.

		*1*. *Feeling understood and supported by health care providers*	*2*. *Having sufficient information to manage my health*	*3*.*Actively managing my health*	*4*.*Social support for health*	*5*.*Appraisal of health information*	*6*.*Ability to actively engage with health care providers*	*7*.*Navigating the healthcare system*	*8*.*Ability to find good health information*	*9*.*Understand health information well enough to know what to do*
Mean score(SD)										
**Age**	<50yrs	2.54(0.77)	**2.29(0.72)**	2.40(0.68)	2.96(0.72)	**2.43(0.76)**	3.49(0.88)	3.15(0.93)	**2.90(1.04)**	**3.35(0.85)**
	≥50yrs	2.43(0.94)	**2.14(0.81)**	2.34(0.86)	2.96(0.64)	**2.25(0.83)**	3.53(1.08)	3.04(1.21)	**2.57(1.17)**	**3.11(0.92)**
	Difference	Put result here	**0.15**	Put result here	Put result here	**0.18**	Put result here	Put result here	**0.33**	**0.34**
	ES*	Put result here	**0.19**	Put result here	Put result here	**0.22**	Put result here	Put result here	**0.31**	**0.27**
**Sex**	Male	2.48(0.88)	2.23(0.81)	**2.45(0.78)**	**3.05(0.70)**	2.33(0.85)	3.45(0.97)	3.06(1.13)	2.76(1.19)	2.28(0.87)
	Female	2.53(0.78)	2.23(0.70)	**2.29(0.72)**	**2.86(0.68)**	2.40(0.73)	3.56(0.94)	3.16(0.93)	2.79(1.00)	3.25(0.90)
	Difference	etc								
	ES*			**0.21**	**0.27**					
**Occupation**	Fishermen	2.49(0.92)	2.17(0.81)	2.42(0.77)	3.0(0.74)	2.3(0.85)	3.42(0.91)	**2.97(1.14)**	2.66(1.1)	3.22(0.83)
	Other	2.51(0.76)	2.28(0.71)	2.33(0.74)	2.9(0.65)	2.41(0.750	3.56(0.99)	**3.21(0.93)**	2.86(1.0)	3.3(0.92)
	Difference	etc						????		
	ES*							**0.23**		
**Income**	≤2000L.E/month	**2.55(0.82)**	2.25(0.74)	2.34(0.72)	2.95(0.68)	2.39(0.76)	3.51(0.95)	3.14(1.01)	2.78(1.08)	3.25(0.87)
	>2000L.E/month	**2.14(0.85)**	2.06(0.88)	2.57(0.95	2.98(0.76)	2.17(0.97)	3.43(0.99)	2.91(1.16)	2.76(1.25)	3.39(0.95)
	Difference	????								
	ES*	**0.49**								
**Internet use**	Use Internet	**2.97(0.87)**	**2.60(0.72)**	**2.62(0.74)**	**3.17(0.92)**	**2.93(0.64)**	**4.05(0.8)**	**3.60(0.73)**	**3.59(0.87)**	**4.07(0.61)**
	Never/rarely	**2.40(0.79)**	**2.15(0.74)**	**2.31(0.75)**	**2.90(0.62)**	**2.24(0.77)**	**3.38(0.85)**	**3.00(1.06)**	**2.60 (1.07)**	**3.08(0.83)**
	Difference	**0.57**	**0.45**	**0.31**	**0.83**	**0.69**	**0.67**	**0.60**	**1.01**	**1.07**
	ES*	**0.70**	**0.60**	**0.41**	**0.54**	**0.92**	**0.79**	**0.62**	**0.95**	**1.34**
**Education**	Illiterate (1)	**2.26(0.88)**	**2.01(0.81)**	**2.33(0.82)**	2.94(0.53)	**2.12(0.85)**	**3.40(1.02)**	**2.79(1.04)**	**2.30(1.05)**	**2.82(0.63)**
	Primary level (2)	**2.61(0.79)**	**2.18(0.66)**	**2.25(0.70)**	2.86(0.76)	**2.32(0.71)**	**3.37(0.99)**	**3.11(1.0)**	**2.74(1.02)**	**3.26(0.96)**
	Higher than primary (3)	**2.69(0.73)**	**2.59(0.66)**	**2.57(0.68)**	3.00(0.77)	**2.75(0.67)**	**3.82 (0.73)**	**3.53(0.79)**	**3.44(0.92)**	**3.86(0.72)**
	Difference (1 vs 2)	**0.35**	**0.17**	**0.08**	Put result here	**0.2**	**0.03**	**0.32**	**0.44**	**0.44**
	ES**	**0.42**	**0.23**	**0.1**	Put result here	**0.25**	**0.02**	**0.31**	**0.42**	**0.54**
	Difference (1 vs 3)	**0.43**	**0.59**	**0.24**	Put result here	**0.63**	**0.42**	**0.74**	**1.14**	**1.04**
	ES**	**0.53**	**0.78**	**0.31**	Put result here	**0.82**	**0.47**	**0.80**	**1.15**	**1.53**

Results in bold have p-value <0.05 for difference in means (tested using robust ANOVA); Effect size (ES) calculated using Cohen’s d for standardized difference in means. Interpretation of ES: “small” ES >0.20–0.50 SD, “medium” ES approximately 0.50–0.80 SD, and “large” ES >0.80 SD.

* ES calculated for statistical significant results

** ES between Illiterate and Non-illiterate groups.

Male participants reported slightly higher scores than women for two scales: *3*. *Actively managing my health* and *4*. *Social support for heath* (ES = 0.21 and 0.27, respectively). Fishing occupation was associated with a lower score for *7*. *Navigating healthcare system* (ES = 0.23) compared with other occupations.

Regarding associations between economic situation and the HLQ, only *1*. *Feel understood and supported by healthcare professionals* indicated that people with low income (below sample median <2000 L.E/month; USD110 on 10 January 2019), reported substantially better than those with a higher income (ES = 0.49).

Across all scales, particpants who used the Internet had higher scores (medium to larg ES) than those who don’t or rarely use the Internet with *8*. *Ability of find good health information* (ES = 0.95) and *9*. *Understand health information well enough to know what to do* (ES = 1.34) showing the largest differences.

We also compared three different levels of education (illiterate, primary, above primary). Differences were found in all the HLQ scales except for scale *4*. *Social support for health*, which was not associated with education level. Across the remaining scales, the largest differences (ES>0.8) were seen for scales *7*. *Navigating the healthcare system*, *8*. *Ability to find good health information* and 9. *Understand health information well enough to know what to do* (see Table 5)

## Discussion

Through the application of a multi-dimensional health literacy questionnaire in a low resource fishing community in Egypt, the abilities of people to understand, access and use health information and health services were determined. Overall, this community had low to very low health literacy, particularly for having enough information, actively managing their health, being able to critically appraise health information, and being able to find good health information. Most participants had some strengths in the areas of social support for health and communicating with health professionals, which reflects a communal and communicative society (i.e., health literacy assets).

Some sub-groups in this Egyptian community reported substantial challenges. Older people had greater challenges with the material elements of health literacy: namely, finding, understanding and appraising health information. Only about one fifth of the respondents used the Internet, and if they did, they had much stronger health literacy on virtually all dimensions. This pattern was striking and indicated that those without access to the Internet were likely to be greatly disadvantaged with regard to accessing health services and effective use of them. Having access to the Internet is related to higher education and literacy, and this is reflected in the strong association between virtually all the HLQ scales and educational achievement. Over one third of the sample were illiterate and this group reported some of the lowest health literacy Unsurprisingly, women were slightly worse with regard to their social support for health and management of their health, which may be due to their husbands being frequently away at work on the lake and being isolated. Most women in this community are engaged in home duties and raising children where it is likely that due to cultural practices, they have limited time to look after themselves and have restricted access to people to look after them when they need help to engage with health practitioners. Futhermore, given their low income and access to money, women tend to spend the money they have on their children rather than on themselves. Other than these differences, men and women had similar health literacy profiles.

In overall, when interpreting the HLQ scores in the fishing villages, most people scored at the ‘disagree’ end of the scales or in the ‘difficult’ to ‘very difficult’ range, including those with higher income or education. Only a small percentage of people with higher than primary education appeared to have a good range of health literacy skills.

While younger people in general are expected to have the capability to search the Internet and find information online [33,34], most people in this study did not engage in Internet searching, or they found it difficult or impossible. This reflects the context of fishing village life, which is a community with poor or no Internet connectivity, a characteristic other rural areas in Egypt [35,36]. Given that the reading ability of most people in this population was very low, even if good quality reading material and/or Internet facilities were available, it would be of limited use.

In our study, fishermen reported slightly lower ability to navigate health services than women. This may be explained by the fact that the fishermen (42% of the study population) face a tight time schedule because of the nature of their job [37]. They have to work long hours at sea and they face many occupational hazards including the physical and psychological strains of shift work and being away from their village. They may not have had opportunities to learn the locations of health services, and may potentially rely on other family members for care and support for their health [38].

Health literacy is a relatively new concept for Egypt, with no previous studies having been conducted using a multi-dimensional health literacy profile tool such as the HLQ. Validity testing of Arabic translation was performed. This type of validity testing is essential when using a health literacy measure developed in a different culture [24]. This is especially important because health literacy is a reflection of an individual’s personal skills but also the complexity of the environment in which, they live, which determines how difficult it is to navigate and access the available health information and services [39]. The issue of the cultural appropriateness of the HLQ and understandability of the concepts was specifically examined in our pre-study work because is an important element of validity evidence for making assertions about what questionnaire data mean [24,40].

The HLQ provides information about potential areas of action and is the basis of the OPtimise HEalth LIteracy and Access (Ophelia) process. The HLQ provides information about potential areas of action and is the basis of the OPtimise HEalth LIteracy and Access (Ophelia) process. The Ophelia approach aimed at identification of health literacy issues specific to the studied community and the development and implementation of specific suitable solutions. Those solutions were directed at organisational, staff, and community member levels. [41]. WHO stated that The Shanghai Declaration of 2016 recognized health literacy as one of the important pillars for achieving the Sustainable Development Goal targets [42]. Interventions should be adopted by communities as well as organizations in orderto achieve improvement in health literacy.

### Strengths and limitations

This the first study to use the widely used HLQ–a psychometrically-robust multi-dimensional measure of health literacy–in a poorly resourced rural community with a high proportion of people who are illiterate. Data from the HLQ has identified several areas of low to very low health literacy which provide guidene on what can be addressed to improve health outcomes. The adapted Arabic HLQ and verbal administration by interviewers enabled members of this rural Egyptian community to record their health literacy needs and so pave the way to take action for more equitable health service resource allocation.

Importantly, our sampling strategy gave opportunity for a wide range of community members to participate, ensuring that even some of the most disadvantaged people in the fishing communities could take part. The HLQ can be administered orally, which means that people do not have to be able to read or write to register their health literacy capacities. This is an important advantage over some other health literacy measures which require respondants to read specific text [43,44]. While this study is not population based (a potential limitation), it does provide a comprehensive profile of the health literacy diversity in the Borollos Lake fishing community, a community that is typical of many communities in Egypt, which reflect communities in many countries in the region.

### Conclusion

This study provided a profile of the vast health literacy challenges, and some health literacy strengths, that people are experiencing in Egypt. The HLQ provides information about potential areas of action and is the basis of the OPtimise HEalth LIteracy and Access (Ophelia) process, a community co-design process for taking action to improve health literacy. The Ophelia process has been adopted by the World Health Organization to support rapid and systematic development of interventions to prevent and control noncommunicable diseases [4,45]. This health literacy work in Egypt is ongoing and includes using the Ophelia process to develop and implement health literacy responsive interventions for fishermen and their families.

## Supporting information

S1 Data (XLSX)
